# Predation shapes the impact of cancer on population dynamics and the evolution of cancer resistance

**DOI:** 10.1111/eva.12951

**Published:** 2020-06-26

**Authors:** Cédric Perret, Cindy Gidoin, Beata Ujvari, Frédéric Thomas, Benjamin Roche

**Affiliations:** ^1^ CREEC/CREES UMR IRD 224‐CNRS 5290‐Université de Montpellier Montpellier France; ^2^ Centre for Integrative Ecology School of Life and Environmental Sciences Deakin University Victoria Australia; ^3^ School of Natural Sciences University of Tasmania Hobart Tasmania Australia; ^4^ Unité mixte internationale de Modélisation Mathématique et Informatique des Systèmes Complexes (UMI IRD/ Sorbonne Université, UMMISCO) Bondy Cedex France; ^5^ Present address: School of Computing, Engineering & Digital Technologies Teeside University Middlesbrough UK

**Keywords:** cancer, cancer resistance, evolution, population dynamics, prey–predator

## Abstract

Cancer is a widespread disease that affects most of the metazoans. However, cancer development is a slow process and, long before causing the death of the individual, may weaken organisms’ capacities and impair their interactions with other species. Yet, the impact of cancer development on biotic interactions, and over the dynamics of the whole ecosystem, is still largely unexplored. As well, the feedback of altered biotic interactions on the evolution of resistance against cancer in the context of community ecology has not been investigated. From this new perspective, we theoretically investigate how cancer can challenge expected interaction outcomes in a predator–prey model system, and how, in return, these altered interaction outcomes could affect evolution of resistance mechanism against cancer. First, we demonstrate a clear difference between prey and predator vulnerability to cancer, with cancer having a limited impact on prey populations. Second, we show that biotic interactions can surprisingly lead to a null or positive effect of cancer on population densities. Finally, our evolutionary analysis sheds light on how biotic interactions can lead to diverse resistance levels in predator populations. While its role in ecosystems is mostly unknown, we demonstrate that cancer in wildlife is an important ecological and evolutionary force to consider.

## 1 INTRODUCTION

Ecological communities are complex systems formed by a large number of interacting organisms, in which the species interplay ultimately affects the stability and functioning of whole ecosystems (Loreau & de Mazancourt, 2013; Thompson, 1999). These interactions are a selective pressure on the life‐history traits of the organisms, which can reciprocally alter the outcomes of the ecological interactions (Van Valen, 1973). In particular, such processes have been extensively studied in prey–predator systems (Abrams, 2000). Nevertheless, there is a constant search for additional factors that can impact eco‐evolutionary feedback loops.

For instance and during the last decade, the fundamental role of parasites in ecosystems has been widely acknowledged (Dobson & Hudson, 1986; Lafferty et al., 2008). Indeed, beyond a direct impact on the mortality of the populations, parasites also strongly interact with other ecological relations by affecting the fitness of their hosts in other ways (Combes, 2001). For example, parasites increase the vulnerability of prey species (Møller & Nielsen, 2007), induce a trade‐off between defence against predator and parasites (Navarro et al., 2004) or weaken the predator's efficacy (Hudson & Greenman, 1998). As a consequence, parasites have been showed to play an important role on the stability and the structure of trophic network (Lafferty et al., 2008; Loreau, Roy, & Tilman, 2007; Thomas, Guégan, & Renaud, 2012), community regulation (Bordes & Morand, 2011) and the risk of population extinction (Macphee & Greenwood, 2013).

While parasites have recently been added to the list of forces structuring ecosystems, it has been suggested that the vast group of cancer diseases should also be considered as selection force (Vittecoq et al., 2013, 2015). Cancer represents a family of diseases characterized by the proliferation of abnormal cells exploiting resources and altering organism health. Apart from being a major public health issue with 19.6% of human deaths worldwide (World Health Organization, 2012), cancer is far from limited to humans. Cancer appeared with multicellularity 100 millions of years ago (Domazet‐Loso & Tautz, 2010; Knoll, 2011) and neoplasia affects the majority of multicellular organism (Aktipis et al., 2015). The lack of appropriate diagnostic tools and the difficulty of detecting cancer in wildlife have led to the widespread view of cancer being a postreproductive disease. However, these conclusions are based on few and weak evidences (McAloose & Newton, 2009; Roche, Møller, DeGregori, & Thomas, 2017), and it is increasingly shown that oncogenic processes are common in wildlife species (Martineau et al., 2002; McAloose & Newton, 2009; Pesavento, Agnew, Keel, & Woolard, 2018; Thomas et al., 2017). For example, cancer prevalence ranges from 1% to 30% among fishes, and up to 25% in birds and mammals (Madsen et al., 2017). Furthermore, cancer prevalence in wildlife is also exacerbated by anthropic activities (Giraudeau, Sepp, Ujvari, Ewald, & Thomas, 2018; Sepp, Ujvari, Ewald, Thomas, & Giraudeau, 2019).

More recently, an increasing amount of evidence suggests that cancer is a gradual phenomenon, potentially beginning early in individual life (Bissell & Hines, 2011; Folkman & Kalluri, 2004; Koebel et al., 2007), opening the possibility that cancer can impact organism's fitness during the reproductive period. Moreover, multiple and diverse cancer resistance mechanisms have been identified (*e.g.* redundancy of tumour suppressor genes, slower somatic mutation rate, different life‐history strategies), providing indirect evidence of the impact of cancer on organism fitness (Caulin & Maley, 2012). The array of such oncogenic phenomena, and any associated resistance, can potentially use up resources and energy, and consequently impact some of the individual's capacities such as vulnerabilities and dispersal (Vittecoq et al., 2013, 2015).

Therefore, conceptually, cancer could play a crucial role in the dynamic interactions of natural communities, which can in turn impact the evolution of organisms by modulating selective pressures for cancer resistance mechanisms (Roche et al., 2017). How the interplay between cancer and biotic interactions can shape population dynamics and the evolution of cancer resistance? To explore this question, we extend a prey–predator model with a possibility of cancer development in order to decipher how oncogenic phenomena can impact the feedback between ecological dynamics and selection for cancer resistance. First, this study aims to investigate whether cancer is a relevant candidate as an ecological factor shaping the population dynamics of wildlife communities. Specifically, we explore how cancer affects the mean densities and the cycles in densities of the predator population and the prey population. Second, this model aims to investigate whether biotic interactions affect the evolution of cancer resistance patterns. To do so, we use the adaptive dynamics framework to simulate the diverse scenario of evolution of cancer resistance while considering the ecological interactions. Overall, this paper presents the first theoretical model to study the interplay between cancer and biotic interaction by explicitly modelling the population dynamics of the interacting species.

## 2 MATERIAL AND METHODS

### 2.1 Background model

We design a theoretical model considering a prey–predator relationship where each species can develop a cancer that alter either its predation capacity or defence against predation capacity. The population dynamics are described by differential equations such as:

(1.1) $$\frac{dP_{h}}{\mathrm{dt}}=e\left[g\left(P_{h},S_{h}\right)+g\left(P_{h},S_{c}\right)+g\left(P_{c},S_{h}\right)+g\left(P_{c},S_{c}\right)\right]-\gamma_{P}P_{h}-\mu_{\mathrm{Ph}}P_{h}$$

(1.2) $$\frac{dP_{c}}{\mathrm{dt}}=\gamma_{P}P_{h}-\mu_{\mathrm{Pc}}P_{c}$$

(2.1) $$\frac{dS_{h}}{\mathrm{dt}}=r\left(S_{h}+S_{c}\right)\left(1-\frac{S_{h}+S_{c}}{K}\right)-g\left(P_{h},S_{h}\right)-g\left(P_{c},S_{h}\right)-\gamma_{S}S_{h}-\mu_{\mathrm{Sh}}S_{h}$$

(2.2) $$\frac{dS_{c}}{\mathrm{dt}}=\gamma_{S}S_{h}-g\left(P_{h},S_{c}\right)-g\left(P_{c},S_{c}\right)-\mu_{\mathrm{Sc}}S_{c}$$

Predator and prey populations are described, respectively, by their densities *P* and *S*. The stages *h* and *c* describe, respectively, healthy and cancerous individuals. Following the mutation accumulation theory (Calabrese & Shibata, 2010; Noble, Kaltz, & Hochberg, 2015; Tomasetti et al., 2015), we assume that cancer develops in healthy population with an occurrence rate *γ*. The fertility of the prey is described by a growth rate *r* and is limited by a carrying capacity *K* of the environment. The fertility of predator population *P* is proportional to the total number of healthy prey captured by healthy predators *g* (*P_h_*, *S_h_*) and by cancerous predators *g* (*P_c_*, *S_h_*), and total number of cancerous prey captured by healthy predators *g* (*P_h_*, *S_c_*) and by cancerous predators *g* (*P_c_*, *S_c_*). This number is modulated by a rate of energetic conversion *e*. The mortality of the prey caused by predation, called extrinsic mortality, is equal to *g* (*P_h_*, *S_h_*) + *g* (*P_c_*, *S_h_*) for healthy individuals and *g* (*P_h_*, *S_c_*) + *g* (*P_c_*, *S_c_*) for cancerous individuals*.* The mortality caused by potential sources of mortality other than predation is called intrinsic mortality. The predator is affected by an intrinsic mortality *µ_P_*. Moreover, and to avoid a bias between population, the prey is also affected by an intrinsic mortality *µ_s_*. The individuals are considered asexual, and we consider only reproductive period. This is a common assumption in population dynamics, made to keep the model tractable. This model does not aim to investigate the role of sexual reproduction, but this factor and its effects could be explored in further work. Growth and mortality depend directly on the densities of prey and predator populations. The predator–prey interaction is described by a function *g*, which is the quantity of prey captured:

(3) $$g\left(P_{i},S_{j}\right)=\frac{P_{i}S_{j}\Phi \left(b_{i},a_{j}\right)}{1+\Phi \left(b_{i},a_{h}\right)S_{h}+\Phi \left(b_{i},a_{c}\right)S_{c}}$$

where *i* and *j* describe either healthy individuals *h* or cancerous individuals *c*. We make the common assumption that the number of prey captured is reduced for high densities of prey because of the time required for predators to process food. Thus, we consider that *g* is a functional response of type II and is described by a logistic relation (Holling, 1959, 1965). The ratio *Φ* between the capacity of predation (*b*) and the capacity of defence against the predation (*a*) modifies directly the number of prey captured:

(4) $$\Phi \left(b_{i},a_{j}\right)=\frac{b_{i}}{b_{i}+a_{j}}$$

### 2.2 Inclusion of oncogenic phenomena

We make the classic assumption that cancer increases the mortality rate of the individuals. However, cancer cells may also reduce the health and the fitness of the cancerous individuals by consuming resources and energy. To understand the potential consequences of these effects, we assume here that capacity of predation (*b*) and capacity of defence against predation (*a*) in addition of intrinsic mortality (*µ*) are affected by oncogenic phenomena such that:

(5.1) $$a_{c}=a_{h}\left(1-\alpha \right)$$

(5.2) $$b_{c}=b_{h}\left(1-\alpha \right)$$

(5.3) $$\mu_{c}=\mu_{h}\;\left(1+\alpha \right)$$

These traits can be assimilated to physiological processes such as run speed, size of the organisms or any specific strategy to hunt or escape (Bolnick et al., 2011), which can be significantly altered by cancer progression. The variable *α* represents the impact on fitness of oncogenic phenomena, which, for simplicity, is assumed linear. As the relation between the progression of cancer and fitness is still unknown and strongly dependent on organs affected (Noone et al., 2017), we will study a whole range of fitness decrease.

### 2.3 Evolution of cancer resistance

We study the evolution of traits that phenotypically modify two main features of cancer resistance: the occurrence rate of cancer *γ* and the cancer's impact on fitness *α*. Both of these features are strongly influenced by the type of cancer and the resistance mechanisms. The occurrence rate *γ* can also be strongly dependent on the ecosystem, *for example* the prevalence of mutagens. We define *α* in [0, 1], which means that *α* can take any value between 0 and 1. Cases with *α* equal to 0 are cases in which cancer does not impact individual characteristics. We define *γ* in [0, 0.1], which means that *γ* can take any value between 0 and 0.1. Cases with *γ* equal to 0 are cases in which there is no cancer within the population. There is limited information on the occurrence rate of cancer in wildlife, and there is a large variation in the prevalence of cancer observed (Madsen et al., 2017). Thus, we do the conservative choice of studying the effect of low to moderate incidence rate and choose an upper bound of 0.1 rather than 1. However, it is important to note that the upper bound chosen can already result in a very high prevalence in the population when the mortality of cancerous individual is low.

**FIGURE 1:**
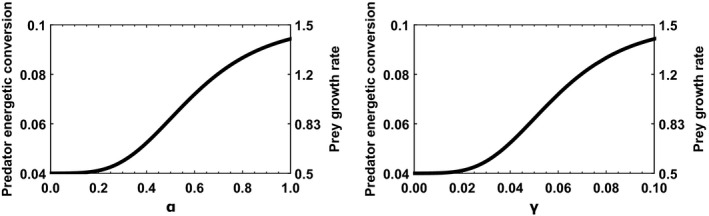
Trade‐off between energetic conversion *e* or the growth rate *r*, and cancer's impact on fitness *α* (on the left) and the occurrence rate of cancer *γ* (on the right). The trade‐off is represented by a Gompertz function such as $y=x_{\min}+\log\left(x_{\max}-x_{\min}\right)+\exp\left(-10*c^{x}\right)$ *. y* describes either the energetic conversion *e* for predators or the growth rate *r* for prey. *x* describes either the cancer's impact on fitness *α* or the occurrence rate of cancer *γ*. For *x = α*, *c = *0.01 and for *x = γ, c = *1.10^−20^. For *y = e, x*_min_ = 0.04 and *x*_max_ = 0.1*.* For *y = r, x*_min_ = 0.1 and *x*_max_ = 1.5

It is worth pointing out that we consider the evolution of resistance mechanisms, which indirectly influence the variables describing cancer, rather than the evolution of cancer properties itself. The variables *α* and *γ* are studied independently, meaning that only one variable is allowed to evolve at a time, while the other is fixed. We assume a cost for the mechanisms of resistance against cancer. This cost is represented by a Gompertz function as shown in Figure 1.

Each trade‐off curve is set to have the inflection point at the median value of the set of possible values of *α* or *γ*. This trade‐off can be explained by the resources required to resist the apparition and progression of cancer but also potential pleiotropic effect of cancer resistance genes. Several trade‐offs between cancer resistance and fitness have been put in evidence (Arnal et al., 2015; Crespi, Summers, Ecology, & Canada, 2006). Since the information on this trade‐off is scarce in the literature, the choice of such function is arbitrary.

### 2.4 Study of the eco‐evolutionary dynamics

The evolution of traits involved in biotic interactions can follow unexpected trajectories because they affect ecological equilibria. For instance, a change in these traits can affect the population densities of prey and predator, which in return affects the fitness value of these traits. To capture these reciprocal interactions, evolution of *γ* and *α* has been simulated using the adaptive dynamic framework (Brannstrom, Johansson, & Festenberg, 2013; Diekmann, 2004). This framework analyses the potential invasion of a monomorphic population carrying a resident trait at equilibrium by a mutant individual carrying a slightly different mutant trait. We make the classic assumptions of adaptive dynamics: ecological dynamics faster than evolutionary dynamics, low mutation rate and small phenotypic effect of mutations (Diekmann, 2004). To study the eco‐evolutionary dynamics of the system, we need to characterize the invasion fitness, which describes the fitness of any mutant trait accordingly to any resident trait. Because of the complexity due to class‐structured populations, we use the approach developed by Hoyle and collaborators, relying on the resident‐mutant system's Jacobian matrix (Hoyle, Best, & Bowers, 2012). While the equation obtained is too complex to be analytically solved, it can nevertheless be solved numerically. Therefore, the expressions of population densities at equilibrium have been approximated through simulation of population dynamics (for further details, see Appendix A).

First, we investigate the effect of cancer on ecological dynamics. To do so, we simulate the densities of the populations of prey and predator using the differential equations presented in Equation (1.1), (1.2), until equilibrium is reached or for 25,000 generations in cases of cycles. The results are represented by bifurcation diagrams showing population densities at equilibrium in case of single‐point equilibrium; and maximum and minimum of population densities in case of limit cycles. Second, we investigate the evolutionary trajectories of resistance against cancer using adaptive dynamics framework. The population densities at equilibrium required for the evolutionary analysis result from similar simulations that in the ecological analysis.

We conduct analysis in three conditions: (1) cancer only present in prey population, (2) cancer only present in predator population, (3) cancer present in prey and predator population. The parameters used for simulations unless specified are *μ_ph_* = 0.01, *μ_sh_* = 0.01, *K* = 4, *a_h_* = 1, *b_h_* = 1. The parameters are chosen in order to maintain coexistence of species while exploring a large range of cancer prevalence as seen in nature (Madsen et al., 2017). The initial population densities of healthy predator and healthy prey are *P_h_* (*t *= 0) = *S_h_* (*t* = 0) = 0.5. There are initially no cancerous individuals in the populations.

## 3 RESULTS

**FIGURE 2:**
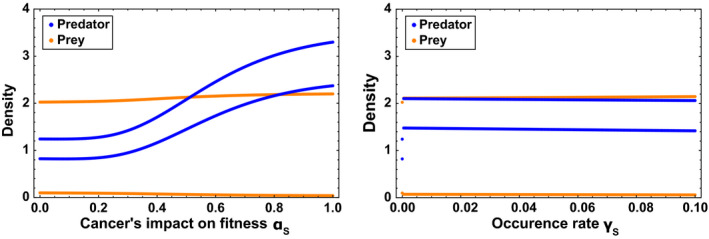
Bifurcation diagram representing the population densities at equilibrium as a function of the cancer's impact on fitness *α_S_* (left) and occurrence rate of cancer *γ_S_* (right). Cancer is present only in the prey population. The predator population and the prey population are, respectively, represented in blue and orange. The default parameters are *α_S_* = 0.5 and *γ_S_* = 0.05

**FIGURE 3:**
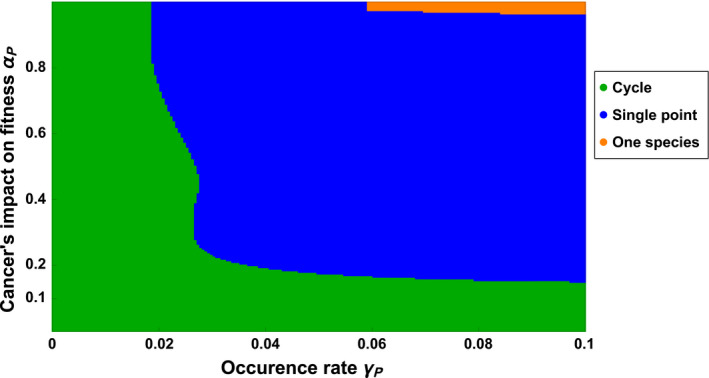
Phase transition diagram representing the community structure as a function of the cancer's impact on fitness *α_P_* and the occurrence rate of cancer *γ_P_*. Cancer is present only in the predator population. The trade‐off considered is between the effect of fitness on cancer *α_P_* and energetic conversion *e*

**FIGURE 4:**
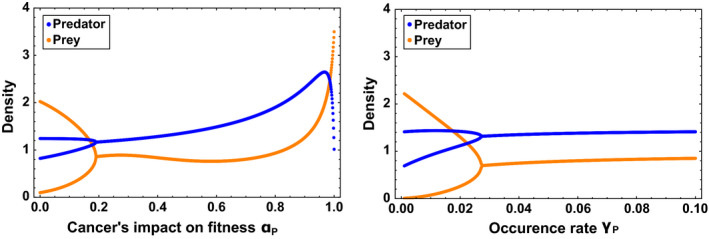
Bifurcation diagram representing the population densities at equilibrium as a function of the cancer's impact on fitness *α_P_* (left) and occurrence rate of cancer *γ_P_* (right). Cancer is present only in the predator population. The predator population and the prey population are, respectively, represented in blue and orange. The trade‐off considered is between the effect of fitness on cancer *α_P_* and energetic conversion *e*. Default parameters are *α_P_* = 0.5 and *γ_P_* = 0.05

The results show a drastic difference in the impact of cancer between prey (Figure 2) and predator populations (Figures 3 and 4). When cancer is present only in prey population (Figure 2), the cancer does not affect the mean prey population density. The only noticeable effect is when cancer has a high impact on organism fitness (*α_s_*), which increases predator population density. This is because cancerous prey have higher susceptibility to predation, which leads to more resources available to predators and thus higher reproduction in the predator population. Importantly, cancer does not affect the community structure and has only a slight effect on the size of cycles. Both species still coexist, and their densities still follow cycles when cancer is present in prey population. This result holds for the entire range of possible values for the occurrence rate of cancer and cancer's impact on fitness.

This absence of effect of cancer in prey population is explained by cancerous prey being quickly removed from the population. First, this is because cancerous prey are subject to higher intrinsic mortality and higher extrinsic mortality. Second, this is because cancerous prey are easier to predate, and thus, cancer in prey increases the density of the predator population. In return, the higher density of predator results in an increase in the mortality due to predation, which mostly affects cancerous prey. The death of cancerous prey reduces the intraspecific competition, increases the reproduction of healthy prey and a fortiori compensates the initially negative effect of cancer. Since the cancer has no effect when present in the prey population, simulations with cancer being present in both species give the same results than simulations with cancer only present in predator population. Thus, only the condition with cancer present in predator population will be extensively investigated.

In contrast to the prey population, cancer strongly affects the ecological dynamics of predator population and can lead to three different community structures, namely (i) coexistence with cycles, (ii) coexistence with single‐point equilibrium and (iii) only prey population present and extinction of predator. Figure 3 presents the effect of the studied variables on the community structure using a phase transition diagram. Figure 3 shows that cancer with low effect on fitness (*α_p_*) or low occurrence rate (*γ_p_*) only reduces the size of the limit cycles at equilibrium without modifying the mean density.

The presence of cancer with a high enough effect on fitness (*α_P_*) and occurrence rate (*γ_P_*) suppresses the cycles and leads populations to single‐point equilibria. Interestingly, the presence of cancer does not affect the mean densities of the populations, while cycles are still present. Instead, an increase of the occurrence rate (*γ_P_*) or the cancer's impact on fitness (*α_P_*) only reduces the size of the cycles. Once the cycles have disappeared, an increase in the cancer's impact on fitness (*α_P_*) of predator leads, first, to an increase in predator density and, second, to a strong reduction of predator density and a high increase in prey density. The second result is not surprising because cancer decreases the fertility and increases the intrinsic mortality of predators. However, the apparent beneficial effect of cancer when cancer has an intermediate effect on fitness hints an important role of the prey–predator dynamics on modulating the effect of cancer on populations.

The results are explained by the fact that cancerous predators are only subject to a higher intrinsic mortality in contrast to the cancerous prey, which are subject to a higher intrinsic mortality and a much higher mortality due to predation. Thus, cancerous predators are maintained in the population. For a low cancer's impact on fitness (*α_P_*), the presence of less efficient predators reduces the predation pressure on prey population and leads to a higher density of prey. In return, it increases the fertility of predators and ultimately compensates the negative effect of cancer. When the effect of cancer on fitness is too high (*α_P_*), cancerous predators do not catch enough prey to sustain themselves. Ultimately, cancerous predators cripple the growth rate of the population and may even lead to the collapse of the population. One could argue that this effect results from the trade‐off between cancer resistance and fertility, or from the effect of cancer on intrinsic mortalities. To investigate the role of each factor, we run simulations excluding either the trade‐off or the effect of cancer on intrinsic mortality (Appendix B). In both cases, the results are qualitatively similar. In addition, simulations considering that the trade‐off is with cancer occurrence rate instead of with the cancer's impact on fitness also produce qualitatively similar results (Appendix C). Therefore, the effect of cancer on the trait involved in the interactions is responsible for the observed impact on the ecological dynamics.

Since cancer affects the ecological dynamics of prey and predators, we now examine whether biotic interactions can affect evolutionary dynamics of resistance against cancer. We restrain our investigation to cases where cancer affects the mean densities of at least one of the two populations. This is because the values of resistance against cancer at equilibrium are not modified when cancer affects only the size of the cycle because in this case, the fitness value of mutant is independent of the value of the resident population. Thus, we consider only the evolution of cancer resistance in the predator population and when single‐point equilibrium is present.

**FIGURE 5:**
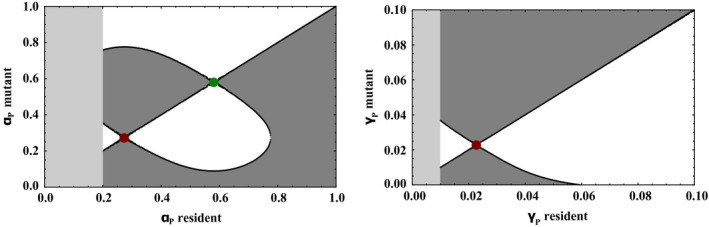
Evolution of the resistance against the cancer's impact on fitness *α_P_* (left) and resistance against the occurrence rate of cancer *γ_P_* (right) in predator population. Pairwise invasibility plot (PIP) represents the potential invasion of an individual carrying a mutant trait in a population of individuals carrying a resident trait. Light grey areas represent parts where cancer does not affect the mean density of population, and thus, evolutionary dynamics are independent of the resident population. Dark grey areas correspond to the values of the mutant trait for which a mutant invades the resident population (invasion fitness superior to 0). Continuously stable strategies (CSSs) are symbolized by a green point and repellors by a red point. Cancer is present only in the predator population

The results are presented graphically by pairwise invasibility plot (PIP) in Figure 5. First, the results of the evolutionary analysis show that two types of evolutionary singular strategies can be observed: continuously stable strategies (CSSs) which are singular strategies convergent and stable; and repellors which are singular strategies divergent and unstable. In other words, the level of resistance will evolve away from the repellor and either towards the CSS or a boundary of the studied range. The choice between these two evolutionary scenarios will depend on the value of the evolving variable in the initial population. For a population with initially high resistance against cancer (low *α_P_*), individuals will evolve an extreme resistance to cancer (*α_P_* = 0). For a population with *α_P_* higher than the value of the repellor, individuals will evolve to an intermediate value represented by the CSS. In addition, the results show that dimorphism is theoretically possible. This coexistence area is illustrated graphically by comparing common invasibility area between the PIP to its symmetric image by the diagonal. However, the dimorphism is converging and would thus disappear in the long term. The presence of diverse evolutionary trajectories is explained by the multiple effects of cancer on the population and its interaction with the prey population.

Figure 5 (right) shows the evolutionary dynamics of the occurrence rate of cancer *γ_P_.* In this case, only one repellor is present. A population with high resistance evolves towards the maximum resistance. A population with low resistance evolves towards the lowest resistance and an occurrence rate of 0.1. The absence of CSS in the occurrence rate *γ_P_* is explained by its limited effect on the population dynamics as shown in the previous section.

## 4 DISCUSSION

Using an extended prey–predator model, we have simulated and analysed (i) the theoretical impact of cancer on biotic interactions and in response (ii) the effect of inter‐ and intraspecific interactions on the evolutionary dynamics of cancer resistance. Our results demonstrate that species interactions can amplify or diminish the impact of oncogenic phenomena, ultimately leading to the evolution of diverse resistance strategies against cancer. First, the model underlines a clear difference between prey population and predator population. Cancer has a limited impact on prey population, and thus, prey population evolves towards an absence of resistance. Second, our results show that cancer with low impact on fitness or incidence can surprisingly have a null or a positive effect on the density of the populations. It also underlies that cycles in population densities limit the effect of cancer, possibly cancel it out when the cancer's impact on fitness is low. Finally, the model sheds light on how biotic interactions can lead to diverse resistance levels in predator population.

These results are explained by the role of biotic interactions, which can amplify or compensate the effects of cancer on the population dynamics. Growth of prey population is limited by (i) two mortality rates, *that is* one intrinsic and one extrinsic due to predation, and (ii) one of these mortality sources is dynamic, *that is* an increase in mortality rate due to predation results in a higher density of predators, which increases again extrinsic mortality rate. As a result, cancerous prey with a higher vulnerability to predation are quickly suppressed from the population. In return, it reduces the intraspecific competition for resources and allows healthy individuals to produce more offspring. Unlike preys, predators are only subject to a fixed intrinsic mortality rate. Thus, the increase in the mortality of cancerous predators is limited and cancerous predators are maintained within the population. For a low effect of cancer, the presence of less efficient predators reduces the predation pressure on prey population and leads to a higher population of prey. In return, the higher density of prey increases the fertility of predators and ultimately compensates the negative effect of cancer. When the effect of cancer is too high, the negative effect of the reduction in the predation capacity overcomes the benefit of higher prey population density. In short, predators do not catch enough prey to sustain themselves. Ultimately, the cancerous predators cripple the growth rate of the population, which may even lead to the collapse of the population.

Predators will evolve towards diverse levels of resistance against cancer as a function of the occurrence rate and the cancer's impact on fitness. Ecological effects make the fitness benefit of resisting cancer more complex with several potential evolutionary trajectory outcomes. It is worth pointing out that we have considered only an effect of cancer on predation capacity. Integrating the impact of cancer on other traits such as dispersion capacity or susceptibility to parasites could lead to a wider diversity of resistance patterns.

This paper presents the first theoretical model to study the interplay between cancer and biotic interaction by explicitly modelling the population dynamics of the interacting species. As discussed in our introduction, our results can be connected to the body of literature looking at the role of parasites (instead of cancer) in prey–predator system (Hatcher, Dick, & Dunn, 2006). Unfortunately, the comparison is limited because (i) these models often work with different assumptions, *for example* infected prey do not reproduce, parasites can be transmitted from prey to predators, and (ii) because parasite populations have their own dynamics which can result in complex effects on the prey–predator dynamic, for example parasites can have a stabilizing (Hudson, Newborn, & Dobson, 1992) or destabilizing effect (Anderson & May, 1986; Ives & Murray, 1997) on a prey–predator system. However, two main results draw interesting parallels with our model. First, the model developed by Packer, Holt, Hudson, Lafferty, and Dobson (2003) found that the preferential hunting of infected prey by predators can cancel the negative effect of parasites and even increase the prey population size (Packer et al., 2003). This result is similar to our results that cancer has a limited effect on prey if it increases the susceptibility of prey to predation. Second, the model developed by Hilker and Schmitz (2008) shows that parasites can stabilize the system, *that is* parasites can reduce the size of cycles in population densities, if parasites induce a higher mortality in predator (Hilker & Schmitz, 2008). Our results reach a similar conclusion for cancer but also show that this effect is amplified if cancer (or parasites) limits the capacity of predators to catch prey.

More generally, this work is in line with other theoretical models investigating the impact of different factors on cancer susceptibility and the evolution of cancer resistance. To date, studies have mainly focused on life‐history traits with or without intraspecific interactions (Aktipis, Boddy, Gatenby, Brown, & Maley, 2013; Boddy, Kokko, Breden, Wilkinson, & Aktipis, 2015; Brown, Cunningham, & Gatenby, 2015; Roche, Sprouffske, Hbid, Missé, & Thomas, 2013). Here, we adopt a complementary approach by considering a similar type of cancer occurring in two interacting natural animal species. For instance, it has been shown how body mass affects the evolution of the activation rate of tumour suppressor genes and oncogenes (Roche et al., 2013). Among others, Roche et al. (2013) have demonstrated the existence of a size threshold above which maintaining high resistance against cancer becomes too costly. We complete this work by showing that integrating the species interactions and their dynamics can lead to more complex evolutionary trajectories. For instance, mass or other factors increasing vulnerability to cancer have potentially a limited effect on the evolution of cancer resistance in prey species. Previous works developed an evolutionary theory (Aktipis et al., 2013) and model (Brown et al., 2015) of cancer resistance which takes life‐history traits into account. The results of Brown et al. (2015) demonstrate that many factors, *for example* life expectancy or reproductive age, affect the cancer incidence and therefore the susceptibility to cancer (Brown et al., 2015). Boddy et al. (2015) have explored the effect of intraspecific competition by integrating reproductive competiveness in a model of the evolution of resistance against cancer (Boddy et al., 2015). Boddy et al. (2015) show that cancer has a low effect in a high extrinsic mortality environment. Our model completes their investigation by explicitly modelling predator population and thus the variation in extrinsic mortality rate of prey population rather than considering a fixed extrinsic mortality rate. In line with their results, our model shows that predation can have a stronger impact on the effect of cancer. Predation can suppress any weakened individuals from the prey population and ultimately negates the effect of cancer.

Despite our model being a simplified version of real ecosystems, it makes some testable predictions. First, it predicts a much lower cancer incidence in prey species that in predator species. If confirmed, it implies that studies on cancer prevalence in wildlife should take into account the interspecific interactions of species considered. Second, our results predict more diverse resistance patterns in predator species. Third, it predicts that cancer with relatively low fitness effect might have a relatively low effect on the densities of populations. To test such predictions, field studies on carcinogenic polluted environment would be a promising path. Yet limited, such studies already suggest that a number of species were hardly affected by the increase of carcinogenic pollution following nuclear catastrophes (Møller & Mousseau, 2006; Mousseau & Møller, 2014). Finally, our model makes the testable prediction that the extinction of predators of a prey population should lead to a dramatic increase in the cancer prevalence in the prey. A similar conclusion was reached in a previous model considering parasitism, which shows that predator removal could dramatically increase the impact of parasites (Packer et al., 2003). On the one hand, this result suggests that the context of predator extinction could be a viable and easier solution to investigate the role of cancer in wildlife. On the other hand, this result suggests that predator control programmes which remove predators to increase prey population size can ultimately be harmful because they would increase the impact of both parasites and cancer on prey.

In this study, we have made a number of assumptions that deserve to be discussed. First, we consider that oncogenic phenomena affect the fitness of individuals since early life stages. This fitness effect is suggested by observations on wildlife populations exposed to carcinogens (Møller & Mousseau, 2006; Mousseau & Møller, 2014) and can be suspected considering that cancer cells (i) consume resources and energy within the individual and (ii) trigger the immune response. Similarly, observations show that parasites, which also exploit host energy and trigger immune response, lead to a higher vulnerability to predators (Hudson, Dobson, & Newborn, 1992; Møller & Nielsen, 2007). Second, we assume a cost of resistance against cancer. This trade‐off has already been assumed in models studying the evolution of resistance against infection (Restif, Hochberg, & Koella, 2004). In addition, limitations between investment in preventing or repairing detrimental mutations and fecundity have been suggested (Moses & Brown, 2003). Third, we have considered that cancer occurs with a certain fixed rate as proposed by the mutation accumulation theory (Calabrese & Shibata, 2010; Noble et al., 2015; Tomasetti et al., 2015). Despite this theory still being discussed, it is considered as the null model for the occurrence of cancer. We also have only considered healthy or cancerous states to keep the model tractable. However, cancer development is a long process which can be described in several stages. Yet, incorporating more stages would not change qualitatively the presented results because cancer would still have a similar negative effect. It is worth noting that a nonlinear development of cancer might lead to more complex evolutionary trajectories and could be explored in future work. Finally, the parameters α and *γ* representing the cancer effect are challenging to assimilate to specific mechanisms. They have been chosen to encompass the main difference between cancer resistance mechanisms, which either offset its apparition or limit its effect (Thomas et al., 2020). An interesting extension would be to refine these effects through different processes and parameters to represent particular mechanisms.

Finally, these results underlie the importance of taking into account the ecological context of wildlife species to understand the evolution of cancer resistance patterns. Further work taking into consideration other interactions such as parasitism (Jacqueline et al., 2017), or other traits affected, could reveal a wide diversity of resistance patterns. Furthermore, additional work could integrate the ecological effect of traits already known to affect cancer incidence. For instance, the size of organisms is correlated to oncogenic phenomena (Nunney & Muir, 2015) but also to the structure of populations (De Roos, Persson, & McCauley, 2003), to the trophic position of species (Cohen, Pimm, Yodzis, & Saldaña, 1993) and to life‐history traits (Woodward et al., 2005).

In conclusion, despite cancer reducing individual capacities, species interactions may compensate or amplify the effect of cancer at the population level. This crucial feedback between ecological dynamics and evolutionary dynamics shapes cancer effect and ultimately cancer resistance patterns. Generally ignored, cancer is now appearing as a potentially important ecological factor for community structure and therefore ecosystem functioning (Roche et al., 2017; Vittecoq et al., 2013). Meanwhile, species interactions also appear as an important factor in the evolution of cancer resistance pattern. The feedback between cancer development and the evolutionary ecology of affected organisms suggests complex evolutionary scenarios and unexpected ecological responses. It is crucial to integrate these dynamics together to understand both sides, in particular in the context of global changes.

## CONFLICT OF INTEREST

None declared.

## Figures and Tables

**FIGURE B1:**
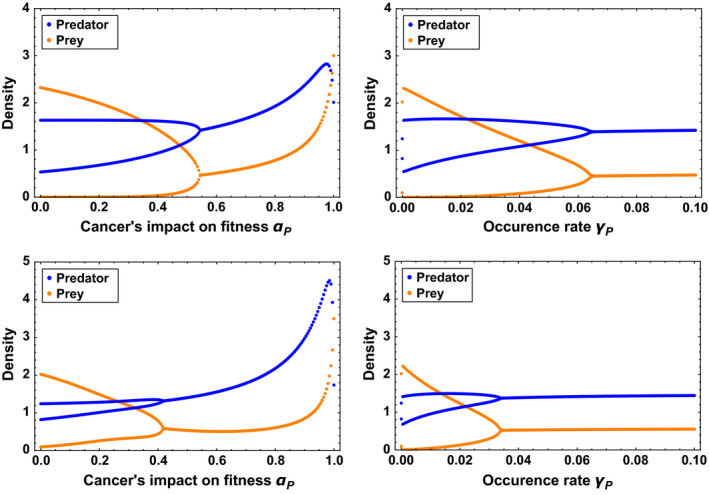
Bifurcation diagram representing the population densities at equilibrium as a function of cancer’s impact on fitness *α_P_* (left) and occurrence rate of cancer *γ_P_* (right) in the absence of trade‐off between resistance against cancer and capacity of predation (top) and in the absence of effect of cancer on intrinsic mortality (bottom). Cancer is present only in the predator population. The predator population and the prey population are, respectively, represented in blue and orange. The trade‐off considered is between the effect of fitness on cancer *α* and energetic conversion. Default parameters are *α_P_* = 0.5 and *γ_P_* = 0.05

**FIGURE C1:**
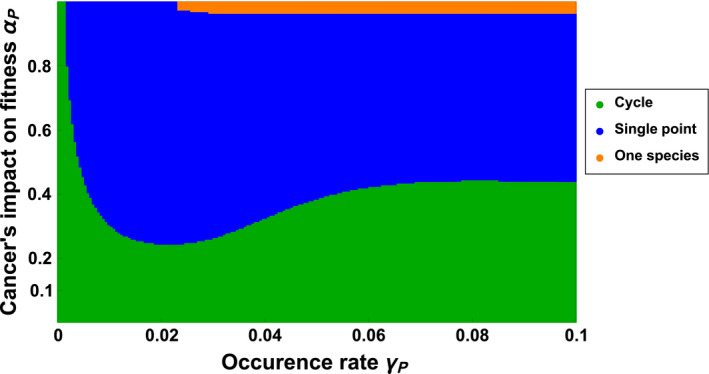
Phase transition diagram representing the community structure as a function of the cancer’s impact on fitness *α_P_* and the occurrence rate of cancer *γ_P_*. Cancer is present only in the predator population. The trade‐off considered is between occurrence rate of cancer *γ_P_* and energetic conversion *e*

**FIGURE C2:**
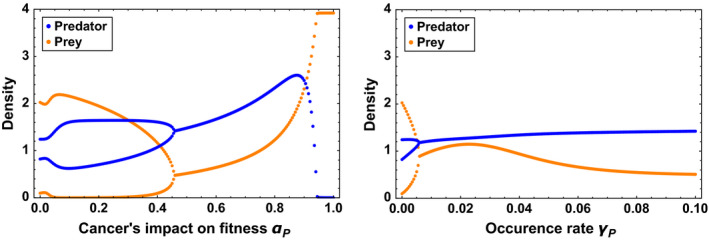
Bifurcation diagram representing the population densities at equilibrium as a function of cancer’s impact on fitness *α_P_* (left) and occurrence rate of cancer *γ_P_* (right). Cancer is present only in the predator population. The predator population and the prey population are, respectively, represented in blue and orange. The trade‐off considered is between the occurrence rate of cancer *γ* and energetic conversion *e*. Default parameters are *α_P_* = 0.5 and *γ_P_* = 0.05

## Data Availability

This manuscript does not use data. The model developed and the specifications of the simulations are fully described in the manuscript.

## Appendix A

### Evolutionary analysis

To study the eco‐evolutionary dynamics of the system, we analyse the potential invasion of a monomorphic population carrying a resident trait *r* at equilibrium by a mutant individual carrying a slightly different mutant trait *m*. To do so, we need to characterize the invasion fitness equation, which describes the fitness of a mutant individual with a trait *m* accordingly to any resident trait *r*. However, in this case, the invasion fitness equation has to describe two classes knowingly the healthy and the cancerous individuals. Because of the complexity of such class‐structured populations, we used the approach developed by Hoyle and collaborators, relying on the resident‐mutant system's Jacobian matrix (Hoyle et al., 2012). To do so, they use the Jacobian matrix which is the square matrix containing the derivative of the two differential equations with respect to the two possible variables:

$$\begin{array}{cc} \frac{\delta \left(P_{H}/dt\right)}{\delta P_{H}} & \frac{\delta \left(dP_{H}/dt\right)}{\delta P_{C}}\\ \frac{\delta \left(dP_{C}/dt\right)}{\delta P_{H}} & \frac{\delta \left(dP_{C}/dt\right)}{\delta P_{C}} \end{array}$$

In the paper of Hoyle et al. (2012), it is shown that the opposite of the determinant of this Jacobian matrix is equivalent to the invasion fitness equation (Hoyle et al., 2012). It is then possible to obtain the invasion fitness equation which is a function of the mutant trait *m* and the values of the population densities at equilibrium of the two classes studied. To obtain the values of the population densities at equilibrium, we simulate the population dynamics until equilibrium is reached. The invasion equation obtained determines whether a mutant individual has higher fitness (invasion), lower fitness (no invasion) or the same fitness (equilibrium) for a given resident trait. We solve the derivative of this fitness equation to determinate the evolutionary singular strategies points. We then look at the second derivative with respect to the resident trait and the second derivative with respect to the mutant trait at the singular strategies previously found. By looking at the sign of each of these derivatives and the sign of their differences, we are able to characterize the singular strategies in regard to their stability. The complete classification of the different evolutionary singular strategies can be found in Diekmann, (2004). For simplicity, we describe only the two types found here knowingly continuous stable strategy (CSS) and repellors. A CSS of value $x_{\mathrm{CSS}}^{*}$ is stable and convergent, which means that (i) a nearby mutant cannot invade a resident population of value $x_{\mathrm{CSS}}^{*}$ , (ii) a mutant individual of value $x_{\mathrm{CSS}}^{*}$ can invade a nearby resident population, and (iii) a resident population *x* can only be invaded by mutants with phenotype closer to $x_{\mathrm{CSS}}^{*}$ than *x* is itself. In other words, evolution would lead to the value of the evolving trait towards a CSS and this value would remain there once reached. A repellor of value $x_{r}^{*}$ is unstable and divergent, which means that (i) a nearby mutant can invade a resident population of value $x_{r}^{*}$ , (ii) a mutant individual of value $x_{r}^{*}$ cannot invade a nearby resident population, and (iii) a resident population *x* can only be invaded by mutants with phenotype further to $x_{r}^{*}$ than *x* is itself. In other words, evolution would lead to the value of the evolving trait away from a repellor.

## Appendix B

### Ecological dynamics of predator population in the absence of trade‐off and in the absence of effect of cancer on intrinsic mortality

To make the distinction between the effect of cancer on the trade‐off and on capacity of predation, and between the effect of cancer on intrinsic mortality and on capacity of predation, we run simulations excluding one or the other of these parameters. In the two cases, the results presented in Figure B1 are qualitatively similar.

## Appendix C

### Ecological dynamics of predator population with trade‐off between occurrence rate of cancer and energetic conversion

Ecological analysis has also been run considering a trade‐off between the occurrence rate of cancer and energetic conversion *e* instead of a trade‐off between the effect of fitness on cancer *α_P_* and energetic conversion e. The results presented in Figures C1 and C2 are qualitatively similar. A low, moderate and high increase in occurrence rate and cancer's impact on fitness leads, respectively, to reduction of the size of cycles, increase of predator population density and decrease of prey population density, and decrease of predator population density (until extinction) and increase of prey population density. A noticeable difference is that for an occurrence rate around 0.4, an increase of the effect of cancer on fitness can first reduce the size of cycle until single‐point equilibrium but then makes appear these cycles again.
